# Circulating tumor DNA as a prognostic biomarker in metastatic colorectal cancer: Post-hoc analysis of treatment response and survival outcomes in the ALTER-C-002 study

**DOI:** 10.17305/bb.2026.13678

**Published:** 2026-02-24

**Authors:** Yue Liu, Jin-Jie He, Sheng Chen, Yun-Jie Song, Jin-Lin Du, Yu-Ping Zhu, Dong-Sheng Chen, Wang-Long Deng, Ying Yuan, Ke-Feng Ding

**Affiliations:** 1 Department of Colorectal Surgery and Oncology (Key Laboratory of Cancer Prevention and Intervention, China National Ministry of Education), The Second Affiliated Hospital, Zhejiang University School of Medicine, Hangzhou, Zhejiang, China; 2 Center for Medical Research and Innovation in Digestive System Tumors, Ministry of Education, Hangzhou, Zhejiang, China; 3 Zhejiang Provincial Clinical Research Center for CANCER, Cancer Center of Zhejiang University, Hangzhou, Zhejiang, China; 4 State Key Laboratory of Neurology and Oncology Drug Development, Jiangsu Simcere Diagnostics Co., Ltd., Nanjing Simcere Medical Laboratory Science Co., Ltd., Nanjing, China; 5 Colorectal Surgery, Zhejiang University Jinhua Hospital, Jinhua, China; 6 Colorectal Surgery, Cancer Hospital of the University of Chinese Academy of Sciences (Zhejiang Cancer Hospital), Hangzhou, China; 7 Medical Oncology, The Second Affiliated Hospital of Zhejiang University School of Medicine, Hangzhou, China

**Keywords:** Circulating tumor DNA, metastatic colorectal cancer, anlotinib, oxaliplatin and capecitabine

## Abstract

Circulating tumor DNA (ctDNA) derived from blood samples can serve as a minimally invasive, real-time indicator of tumor burden and treatment response in metastatic colorectal cancer. This study evaluated the association between baseline ctDNA levels and changes in ctDNA during treatment with response and survival outcomes in the phase II ALTER-C-002 trial, which investigated first-line anlotinib combined with capecitabine and oxaliplatin in patients with rat sarcoma (RAS) and B-Raf proto-oncogene (BRAF) wild-type metastatic colorectal cancer. In this post-hoc biomarker analysis, plasma samples were collected at baseline, near the best radiologic response (C1), and at the timepoint closest to disease progression or last follow-up (C2). These samples were analyzed using a 90-gene next-generation sequencing (NGS) panel, with ctDNA abundance quantified as maximum somatic allele frequency (MSAF), where MSAF <0.001 indicated ctDNA negativity. Biomarker data were obtained for 26 patients, 25 of whom had measurements at all three timepoints. Notably, baseline MSAF was higher in patients with liver-only metastasis (*P ═* 0.017). MSAF decreased significantly at C1 compared to baseline (*P <* 0.001) and increased at C2 relative to C1 in patients exhibiting progressive disease (PD) (*P <* 0.001); additionally, higher MSAF at C1 was associated with subsequent PD at C2 (*P ═* 0.015). All patients who tested ctDNA-negative at C1 achieved an objective response, and ctDNA negativity at this timepoint correlated with longer progression-free survival (PFS) (*P ═* 0.012) and overall survival (OS) (*P ═* 0.006) compared to ctDNA-positive patients. Furthermore, KRAS/BRAF mutations detected in ctDNA were linked to shorter PFS and OS (all *P <* 0.05). In conclusion, baseline ctDNA burden and early ctDNA clearance may function as prognostic and on-treatment biomarkers of therapeutic efficacy in metastatic colorectal cancer, warranting prospective validation.

## Introduction

Colorectal cancer (CRC) accounts for approximately 10% of all cancer cases and is the second leading cause of cancer-related mortality, with over 1.85 million cases and an estimated 0.85 million deaths annually [1, 2]. The global incidence of CRC is on the rise [3]. This increasing occurrence has been partially attributed to a greater understanding of cancers of unknown primary (CUP), which include certain subsets of CRC recently identified [4]. Among new CRC diagnoses, 20% of patients present with metastatic disease, while an additional 25% with localized disease will eventually develop metastases [5]. Systemic therapy, which includes cytotoxic chemotherapy, biological therapy targeting cellular growth factors, immunotherapy, and their combinations, remains the primary treatment for metastatic CRC (mCRC). Unfortunately, the prognosis is poor, with a 5-year survival rate of approximately 14% [5–7]. Given that CRC is predominantly an age-associated malignancy, with nearly 70% of cases diagnosed in individuals over 65, prognosis in older patients may be complicated by variations in disease stage at diagnosis, tumor location, preexisting comorbidities, and the nature of treatments received [8].

Biomarkers indicating treatment outcomes and disease progression are crucial for clinical decision-making. Recent studies have demonstrated that the mutational status of these biomarkers, such as RAS mutations and mismatch repair (MMR) deficiency or microsatellite instability-high (MSI-H) status, correlates with survival benefits for patients with mCRC [9, 10]. Despite these advancements, the clinical utility of biomarkers in mCRC remains limited [11, 12]. Currently, detection of defective DNA mismatch repair primarily utilizes immunohistochemistry (IHC) and/or MSI testing. However, translating MSI/MMR status into actionable clinical information is hindered by biological and technical variability. Discrepancies in IHC-based MMR assessments have been observed for identical germline mutations, potentially due to additional somatic alterations, complicating biomarker interpretation and clinical decision-making [13].

Circulating tumor DNA (ctDNA) is a subset of total cell-free DNA (cfDNA) found in the bloodstream, enabling real-time evaluations of patient prognoses and treatment responses through simple blood draws [14, 15]. Several studies have highlighted the potential of ctDNA in guiding targeted treatment strategies for specific mCRC subtypes [16, 17]. Additionally, ctDNA has been shown to influence treatment outcomes for mCRC in several investigations [18–20]. The measurement of ctDNA abundance, typically represented by maximum somatic allele frequency (MSAF), has been proposed as a means to estimate tumor content [21]. The ALTER-C-002 study, a phase II, multicenter, single-arm trial assessing the efficacy and tolerability of anlotinib in combination with capecitabine and oxaliplatin (CAPEOX) for first-line therapy in RAS/BRAF wild-type mCRC, demonstrated promising efficacy with manageable toxicity profiles [22]. Consequently, further investigation of dynamic ctDNA changes is warranted to explore their potential associations with clinical outcomes in mCRC patients undergoing systemic therapies. This post-hoc analysis aims to examine the correlations between ctDNA levels and patient outcomes, alongside reporting overall survival (OS) data from the ALTER-C-002 trial.

## Materials and methods

### Study design and participants

This analysis included 30 patients from the phase II, single-arm, multicenter trial (ALTER-C-002), which investigated the efficacy and safety of anlotinib combined with CAPEOX as first-line treatment for mCRC. Detailed trial information is available at ClinicalTrials.gov (identifier: NCT04080843) [22]. Eligible patients were aged 18–75 years with histologically or cytologically confirmed RAS/BRAF wild-type mCRC, determined via polymerase chain reaction (PCR) or next-generation sequencing (NGS), and an Eastern Cooperative Oncology Group performance status (ECOG PS) of 0 or 1. Patients with RAS/BRAF wild-type mCRC who had not received prior systemic therapy or who had previously undergone neoadjuvant/adjuvant chemotherapy for stages I-III CRC, and who experienced relapse more than 6 months after the last administration of perioperative chemotherapy, were included. Treatment consisted of capecitabine (850 mg/m^2^, oral, twice daily, on days 1–14), oxaliplatin (130 mg/m^2^, intravenous, on day 1), and anlotinib (12 mg, oral, once daily, on days 1–14), administered in 21-day cycles for a total of 6 cycles.

The ALTER-C-002 study protocol received ethical approval (approval number: No.2019 [268]) from the central ethics committee of the Second Affiliated Hospital of Zhejiang University School of Medicine, Zhejiang Cancer Hospital, and Zhejiang University Jinhua Hospital. The study was conducted in accordance with the principles of the Declaration of Helsinki, Good Clinical Practice Guidelines, and local regulations. Written informed consent was obtained from all participants prior to plasma sample collection.

Plasma samples were collected at baseline (1 week prior to treatment), C1 (close to optimal remission, within 1 week before or after imaging assessment confirming the best overall tumor response), and C2 (at the data cutoff; for patients with progressive disease [PD], the sample closest to radiologic progression within ±1 week of imaging; for patients without PD, the last follow-up sample before data cutoff).

### ctDNA assays

cfDNA was extracted from plasma using the Magbead Free-Circulating DNA Maxi Kit (CW2560S, CWBIO) on the AE2130 Nucleic Acids Extraction Automation System (CWBIO), following the manufacturer’s instructions. Genomic DNA (gDNA) was extracted from a 200 µL buffy coat sample using the Blood gDNA Extraction Kit (CW2361S, CWBIO) on the AE2130 system for mutation calling from ctDNA.

Library preparation for gDNA was performed using the VAHTS Universal Plus DNA Library Prep Kit (Vazyme, Cat. ND617) according to the manufacturer’s guidelines. Briefly, white blood cell (WBC) gDNA samples were fragmented to a peak size of 200 bp using the FEA enzyme mix. The fragmented DNA underwent end-repair and dA-tailing, followed by ligation with universal adapters. After post-ligation cleanup, the ligated products were PCR amplified with index primers. cfDNA library preparation was conducted using the KAPA Hyper Preparation Kit (Kapa Biosystems, Wilmington, MA, USA) according to the manufacturer’s recommendations. In this process, up to 80 ng of extracted cfDNA samples were end-repaired, followed by ligation with adapters containing unique molecular identifiers (UMIs). Post-ligation cleanup was performed using AMPure XP Beads (Beckman Coulter, Cat. A63882), and the ligated products were PCR amplified with dual-indexed primers. A minimum of 500 ng of each library was hybridized and captured using a fixed tumor-agnostic 90-gene panel for 12–16 h. Target enrichment was achieved using the xGen™ Hybridization and Wash kit, following the manufacturer’s instructions. Sequencing was conducted on a NovaSeq 6000 sequencer (Illumina, San Diego, CA, USA) with 2 × 151 bp paired-end reads and unique dual indexing, achieving a mean target coverage of 30,000 × for cfDNA and 6,000 × for gDNA samples.

Preliminary sequencing results in BCL format were converted to FASTQ files using bcl2fastq (v2.20.0) [23]. After filtering out adaptors and low-quality reads, clean FASTQ data were aligned to the human reference genome (hg19) using the BWA-MEM aligner (v.0.7.17) [24] with default parameters. Additional realignment of select regions was conducted using ABRA2 (v.2.21) [25]. Candidate tumor-specific mutations, including point mutations, small insertions, and deletions, were identified and annotated using VarDict (v.1.5.7) [26] and InterVar [27]. To enhance analytical specificity and minimize confounding from clonal hematopoiesis (CH) and technical artifacts, matched WBC gDNA was utilized as a paired normal control, and an in-house pipeline incorporating UMI-based error correction and empirical CH filtering was employed. A detailed analysis of ctDNA is available in the supplementary materials.

### ctDNA dynamic monitoring

In this cohort, ctDNA detection was conducted using a 90-gene tumor-agnostic panel across baseline, C1, and C2. The fixed panel did not include a tumor-informed component. ctDNA data were available for 26 patients at baseline and C1. The mutational profile was analyzed in these 26 patients with baseline ctDNA information. An analysis of ctDNA kinetics was performed for 25 patients (one patient was excluded due to an unavailable ctDNA sample at C2) who had ctDNA data at all three timepoints (baseline, C1, and C2).

MSAF, a method for estimating the fraction of tumor-derived ctDNA relative to total cfDNA, was calculated for all samples. This metric provides an estimate of the ctDNA fraction present in the blood. The MSAF calculation was based on cfDNA data, with WBC and gDNA data used for filtering germline mutations and CH. MSAF was determined by calculating the allele fraction (AF ≥ 0.1%) for single nucleotide variants (SNVs) and insertions/deletions (InDels) per sample, excluding rearrangements and copy number alterations (CNAs); the maximum AF measured is defined as MSAF. An MSAF value of <0.001 indicated a ctDNA-negative sample [28, 29].

### Assessments and statistical analysis

Tumor response, survival evaluation (progression-free survival [PFS]), clinical endpoint definitions, and statistical approaches for each endpoint have been previously published [22]. Briefly, tumor response was assessed based on the investigator’s evaluation according to the Response Evaluation Criteria in Solid Tumors (RECIST) version 1.1, conducted via computed tomography or magnetic resonance imaging during the screening period and every two cycles throughout the study until disease progression. OS was defined as the time from the first dose of the study drug to death from any cause and was calculated with reference to PFS. For baseline ctDNA-based stratification, PFS and OS were calculated from the date of the first dose of the study drug, including all enrolled patients.

For biomarker analysis at baseline, the mutational landscape was evaluated, and the relationship between ctDNA levels and clinicopathologic factors (metastatic site, tumor location, and primary resection status) was assessed. Additionally, we analyzed the impact of ctDNA levels on efficacy in various subgroups: patients with PD vs non-PD (classified according to tumor response at the C2 timepoint), ctDNA-positive patients vs ctDNA-negative patients at the C1 timepoint, high-MSAF vs low-MSAF (using the median value as the cutoff) at baseline, and KRAS/BRAF mutations vs wild-type patients. Categorical variables were summarized as frequencies (percentage [%]), while continuous variables were presented as medians with ranges. The non-parametric Wilcoxon test was utilized to compare continuous ctDNA-related variables. A paired Wilcoxon signed-rank test was performed to compare dynamic changes of ctDNA across three timepoints in 25 patients throughout the ctDNA testing process, while an unpaired Wilcoxon rank-sum test was conducted for between-group comparisons at a given timepoint. Multiple longitudinal comparisons were corrected using the Bonferroni test. Survival curves were compared using the log-rank test. The association of ctDNA with PFS and OS was assessed using Kaplan–Meier curves. Multivariable Cox regression analyses were conducted for hypothesis generation, incorporating ctDNA status and adjusting for key clinically relevant covariates, including age, gender (female vs male), presence of liver metastasis only (No vs Yes), and tumor location (left-sided vs right-sided) [30, 31]. Gene alteration landscapes were visualized using the Complex Heatmap package (2.12.0), and Kyoto Encyclopedia of Genes and Genomes (KEGG) pathway analysis of identified gene alterations was performed using the ClusterProfiler package (v 4.4.4). All statistical analyses were conducted using SPSS software (version 26.0, SPSS Institute, IL, USA), GraphPad Prism (version 6.00, GraphPad Software), and R system (version 4). A *P* value <0.05 was deemed statistically significant.

## Results

### Patient characteristics and clinical outcomes

Among the 30 patients with mCRC enrolled in the ALTER-C-002 study, the majority were male (26/30, 86.7%) and had an ECOG performance status score of 1 (27/30, 90.0%), with a median age of 60 years (range 32–72). By the cutoff date for the updated efficacy analysis (May 2023), with a median follow-up of 31.7 months (95% CI, 27.8–35.6), the OS for all patients (*n* ═ 30) was reported as a median of 29.7 months (95% CI, 21.16–38.24; Figure S1A). Among the 26 patients with analyzable biomarker data, the median OS was 29.7 months (95% CI, 27.46–31.94), and the median PFS was 11.3 months (95% CI, 7.3–14.52; Figure S1B and C); the overall response rate (ORR) was 76.9% (95% CI, 56.4%–91.0%), which included one complete response (CR) and 19 partial responses (PR).

**Figure 1.**
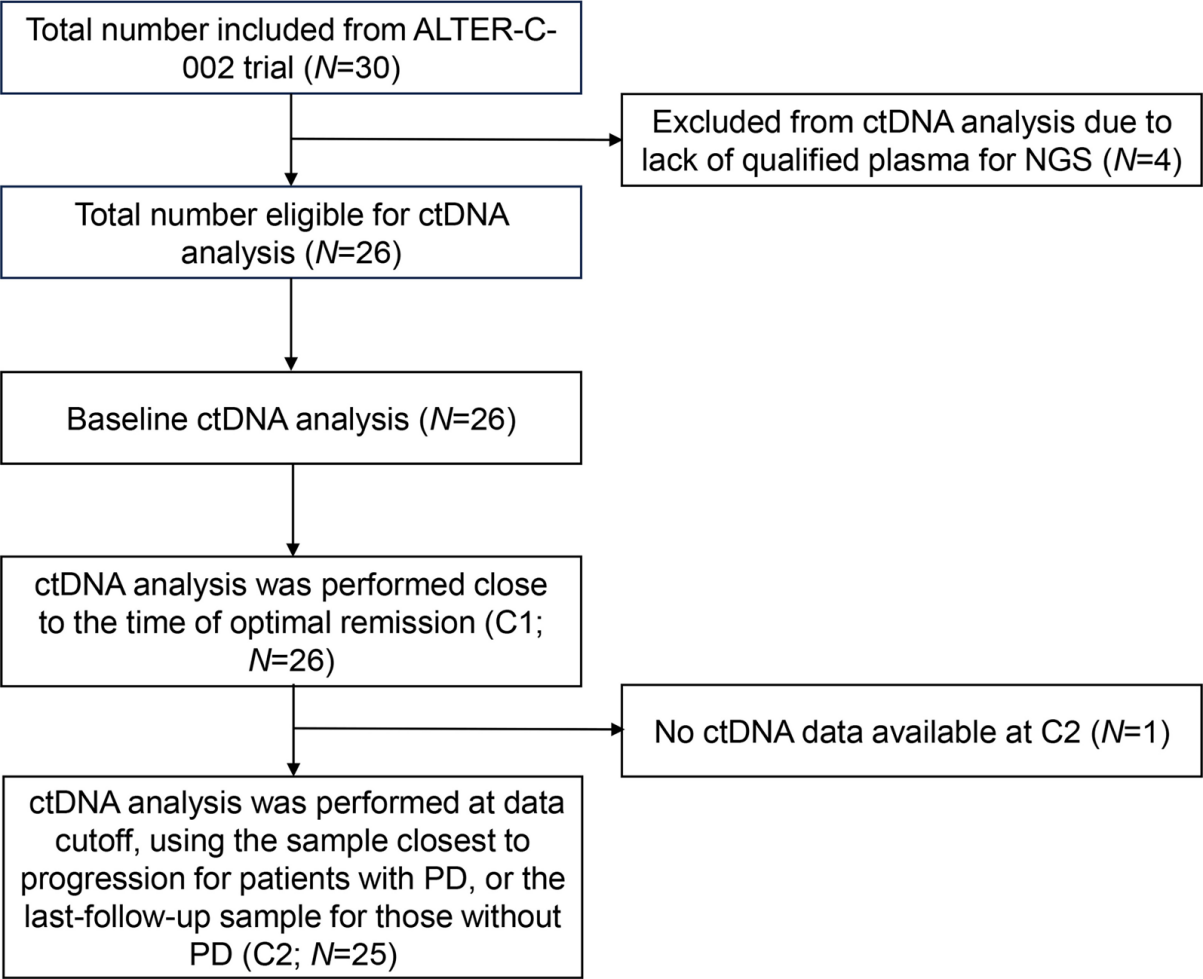
**Study design and timing of ctDNA collection.** Abbreviations: ctDNA: Circulating tumor DNA; NGS: Next-generation sequencing; PD: Progressive disease.

Plasma samples were available for a total of 26 patients, with four patients excluded from the analysis due to the lack of qualified plasma for NGS (Figure 1). A total of 77 plasma samples from the 26 patients with mCRC were utilized for ctDNA analysis.

### Mutation landscape in baseline ctDNA

**Figure 2.**
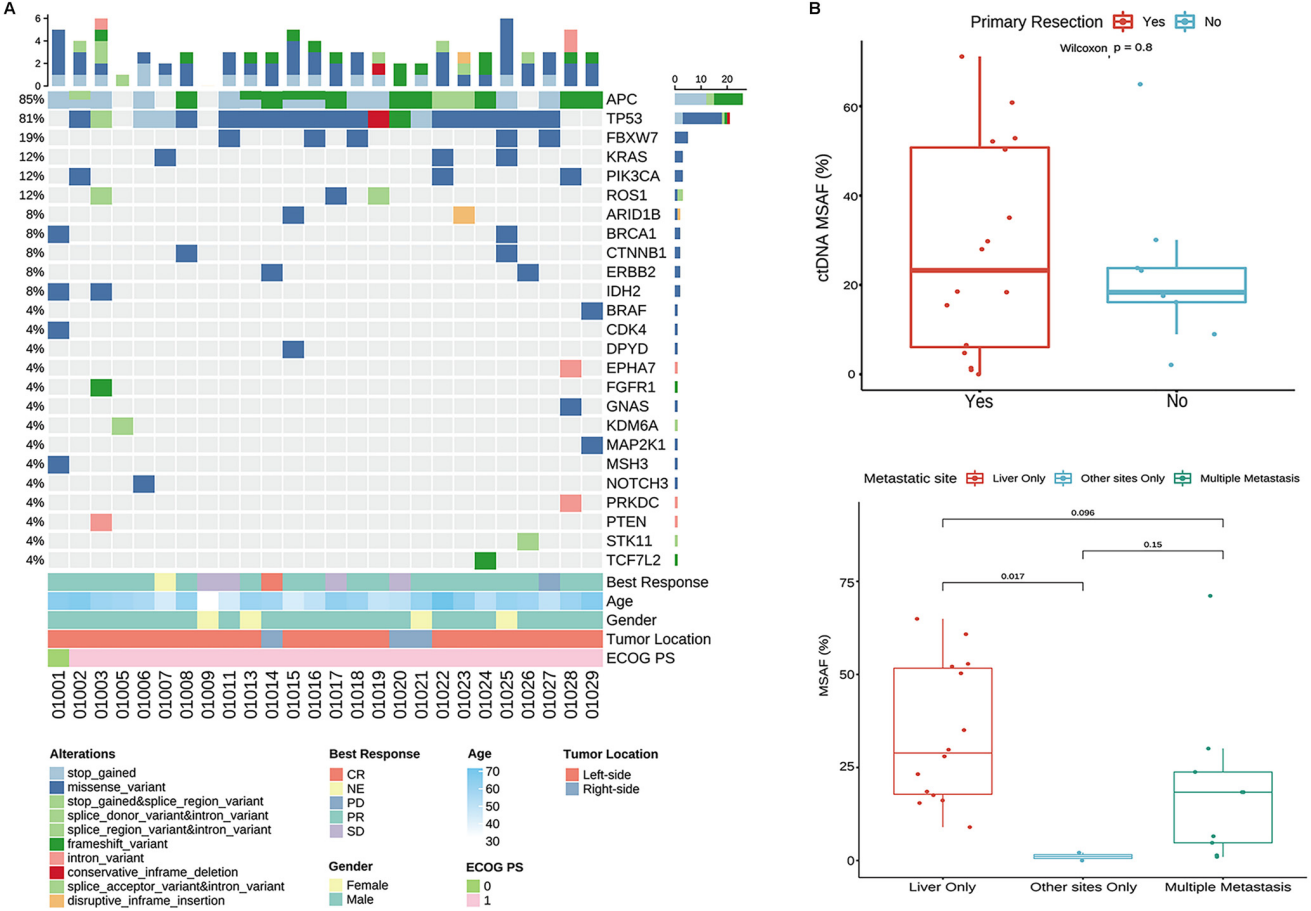
**Baseline circulating tumor DNA genomic landscape and association of baseline ctDNA burden with clinicopathologic factors.** (A) Oncoprint summarizing somatic alterations detected in baseline plasma ctDNA using a tumor-agnostic 90-gene NGS panel in 26 patients; each column represents an individual patient and each row a gene (genes shown are those altered in at least one sample). Colored tiles indicate variant consequence classes as shown in the key, with bar plots summarizing the number of alterations per patient (top) and alteration frequency by gene (side). Clinical annotations (best overall response, age, sex, primary tumor sidedness, and Eastern Cooperative Oncology Group performance status) are shown below the oncoprint. (B) Baseline ctDNA burden quantified as MSAF compared by primary tumor resection status (Yes, *n* ═ 17; No, *n* ═ 9) and metastatic site (liver only, *n* ═ 15; other sites only, *n* ═ 2; multiple metastases, *n* ═ 9). Box plots display median and interquartile range, with whiskers indicating 1.5× the interquartile range and points representing individual patients. Unpaired Wilcoxon rank-sum tests were used to generate the *P* values (pairwise comparisons as indicated); baseline MSAF was higher in patients with liver-only metastasis versus other-sites-only metastasis (*P ═* 0.017). Abbreviations: ctDNA: Circulating tumor DNA; ECOG: Eastern Cooperative Oncology Group; MSAF: Maximum somatic allele frequency; NGS: Next-generation sequencing; PD: Progressive disease; PR: Partial response; CR: Complete response; SD: Stable disease; PS: Performance status.

Baseline ctDNA profiles of alterations in 90 genes were obtained from blood samples collected from 26 patients in this trial (Figure 2A). The baseline ctDNA positivity rate was 96.2% (25/26), with the highest mutation frequencies observed in the adenomatous polyposis coli (*APC*; 84.6%, 22/26) gene, followed by tumor protein p53 (*TP53)* (80.8%, 21/26), F-box and WD repeat domain containing 7 (*FBXW7)* (19.2%, 5/26), *KRAS* (11.5%, 3/26), phosphatidylinositol-4,5-bisphosphate 3-kinase catalytic subunit alpha (*PIK3CA)* (11.5%, 3/26), and ROS proto-oncogene 1, receptor tyrosine kinase (*ROS1)* (11.5%, 3/26; Table S1). Genotyping of baseline ctDNA captured intrapatient genomic heterogeneity, including mutations in *KRAS* and *BRAF*. Of the patients carrying mutations in *KRAS* and *BRAF*, a majority (75%, 3/4) achieved PR following treatment with anlotinib plus CAPEOX.

Baseline MSAF levels were significantly associated with the metastatic site; specifically, the median MSAF was significantly higher in patients with liver metastasis only compared to those with other metastatic sites (*P ═* 0.017; Figure 2B). However, baseline MSAF levels were not associated with any other baseline clinicopathologic factors, including tumor location, primary resection status, or the presence of multiple metastases compared to other metastatic sites (all *P* > 0.05; Figure 2B).

### Dynamics of ctDNA during treatment

**Figure 3.**
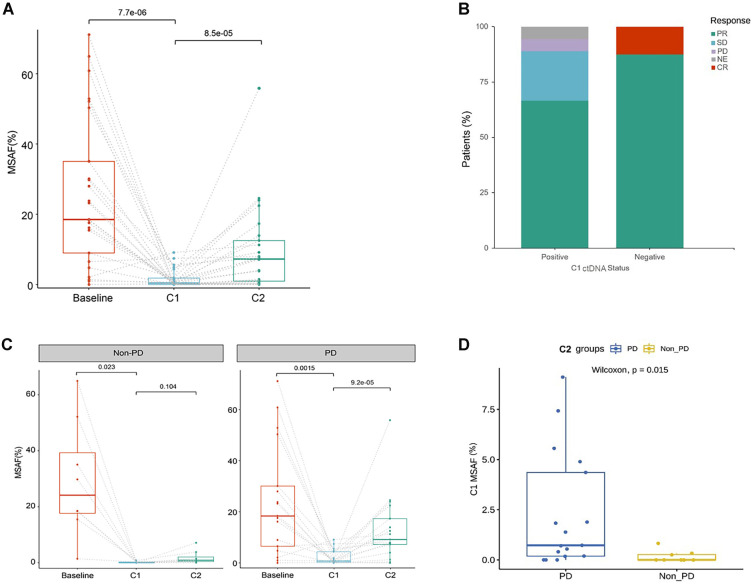
**Longitudinal circulating tumor DNA kinetics and association with radiologic response during first-line anlotinib plus capecitabine and oxaliplatin.** (A) Plasma ctDNA abundance, quantified as MSAF, measured at baseline (pre-treatment), C1 (timepoint near best radiologic response), and C2 (sample closest to radiologic progression or last follow-up) in patients with complete sampling (*n* ═ 25). Individual patient trajectories are connected by dashed lines; box plots display median and interquartile range. Paired Wilcoxon signed-rank tests with Bonferroni correction were used for the baseline–C1 and C1–C2 comparisons, and the resulting *p* values are shown above the brackets. (B) Distribution of best overall response assessed by RECIST version 1.1 according to C1 ctDNA status, defining ctDNA negativity as MSAF <0.001 (ctDNA-positive, *n* ═ 17; ctDNA-negative, *n* ═ 8); categories include partial response, stable disease, progressive disease, complete response, and not evaluable. (C) MSAF trajectories stratified by C2 outcome, comparing patients with progressive disease (PD; *n* ═ 17) versus those without progression (non–PD; *n* ═ 8); within-group longitudinal comparisons use paired Wilcoxon signed-rank tests with Bonferroni-adjusted *P* values (shown). (D) Comparison of C1 MSAF between patients classified as PD versus non–PD at C2 using an unpaired Wilcoxon rank-sum test (*P ═* 0.015; unadjusted). Abbreviations: CR: Complete response; ctDNA: Circulating tumor DNA; MSAF: Maximum somatic allele frequency; NE: Not evaluable; PD: Progressive disease; PR: Partial response; RECIST: Response Evaluation Criteria in Solid Tumors; SD: Stable disease.

We investigated the correlation between ctDNA dynamics during treatment and patient response. A total of 25 patients with complete ctDNA testing at three time points were analyzed for dynamic changes in MSAF during treatment, while one of 26 patients with an unavailable ctDNA sample at C2 was excluded. The results indicated a significant decrease in MSAF levels at C1 compared to baseline (*P <* 0.001, Figure 3A). Notably, all 8 patients who were ctDNA-negative achieved an objective response (CR=1; PR=7), whereas 12 of the 17 patients in the ctDNA-positive group achieved a PR (Figure 3B).

At the C2 timepoint, 17 (68%) patients experienced PD, while 8 (32%) remained non-PD. The median MSAF level was significantly higher at C2 than at C1 in the PD group (*P <* 0.001); however, no significant differences were noted between C2 and C1 in the non-PD group (all *P ═* 0.104, Figure 3C). Further analysis of MSAF levels at C1 revealed that the median was significantly higher in patients with PD compared to those who were non-PD (*P ═* 0.015, Figure 3D).

**Figure 4.**
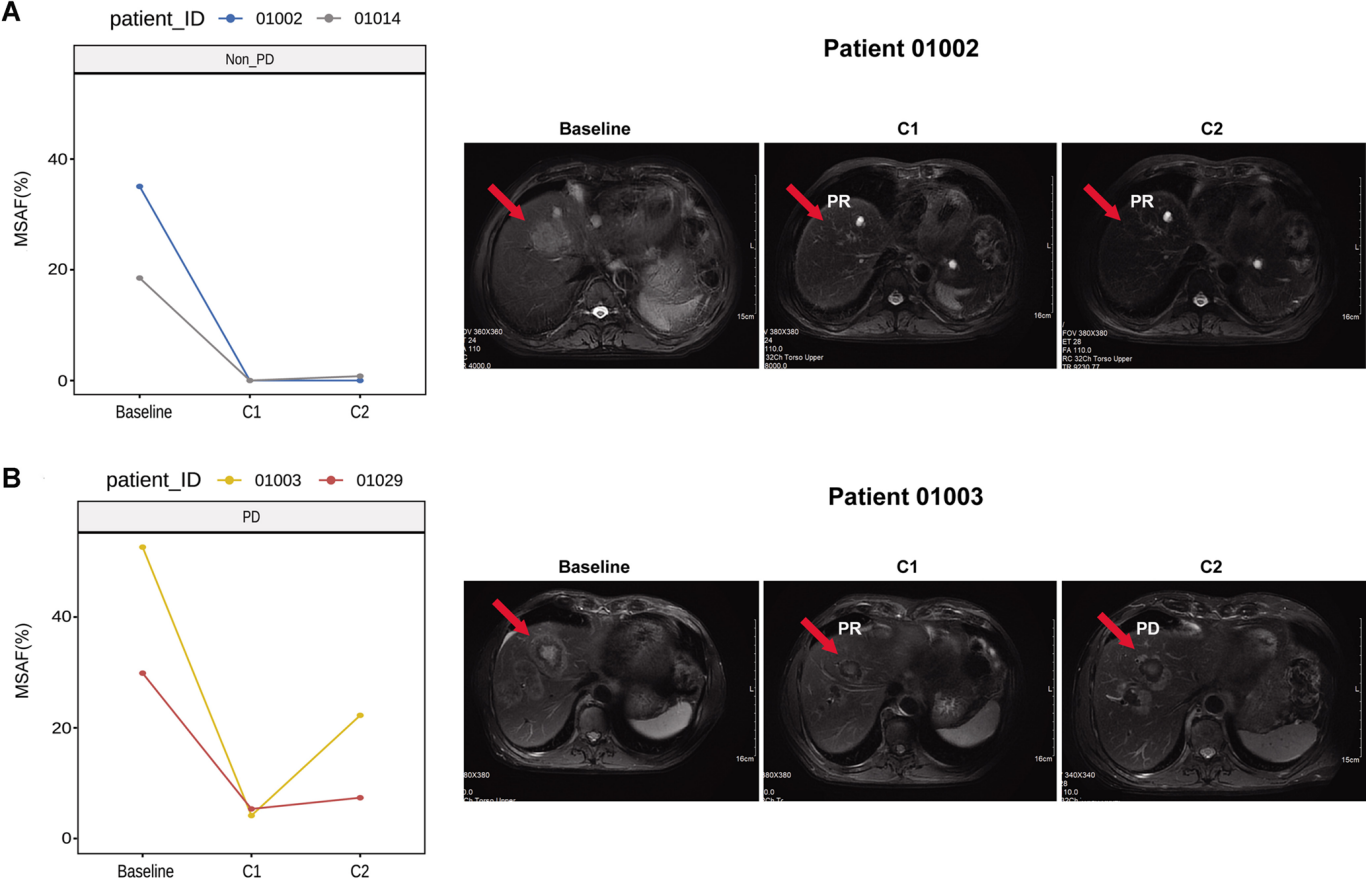
**Representative longitudinal changes in circulating tumor DNA burden and radiographic tumor dynamics during treatment.** (A) Two patients classified as non-PD at C2 (patient IDs 01002 and 01014) showing a marked decline in MSAF from baseline to C1 with sustained low/undetectable levels at C2 (left; MSAF <0.001 indicates ctDNA negativity). Representative radiographic images for patient 01002 (right; red arrows indicate target lesions) illustrate partial response at C1 that was maintained at C2. (B) Two patients classified as PD at C2 (patient IDs 01003 and 01029) showing an initial decrease in MSAF at C1 followed by an increase at C2 (left). Representative radiographic images for patient 01003 (right; red arrows indicate target lesions) demonstrate partial response at C1 followed by PD at C2. C1 denotes the timepoint near best radiologic response, whereas C2 denotes the sample closest to radiologic progression for PD patients or the last follow-up sample for non–PD patients; responses were assessed according to Response Evaluation Criteria in Solid Tumors version 1.1. Abbreviations: C1: Timepoint near best radiologic response; C2: Timepoint closest to radiologic progression or last follow-up; ctDNA: Circulating tumor DNA; MSAF: Maximum somatic allele frequency; non–PD: Non–progressive disease; PD: Progressive disease; PR: Partial response; RECIST: Response Evaluation Criteria in Solid Tumors.

We also described the dynamic changes in ctDNA and tumor volumes during treatment in four representative cases. For instance, patient ID 01002 exhibited ctDNA-negativity (MSAF value of <0.001) and a PR at the C1 timepoint, which was maintained at C2 (Figure 4A). In contrast, patient ID 01003, who presented with a PR at C1, exhibited a decreasing trend in MSAF levels, with an increase observed upon progression to PD at C2 (Figure 4B).

To further elucidate the genetic dynamics, we examined the distribution of mutations across KEGG pathways in the PD group. The most frequent mutations identified were *APC* (82.35%), *TP53* (76.47%), and *FBXW7* (23.53%), which are involved in the WNT, TP53, and Notch pathways (Figure S2). Additionally, mutations in *KRAS* and *BRAF*—likely drivers of resistance to epidermal growth factor receptor (EGFR) antibodies—were detected in two patients with PD.

### Survival analysis

**Figure 5.**
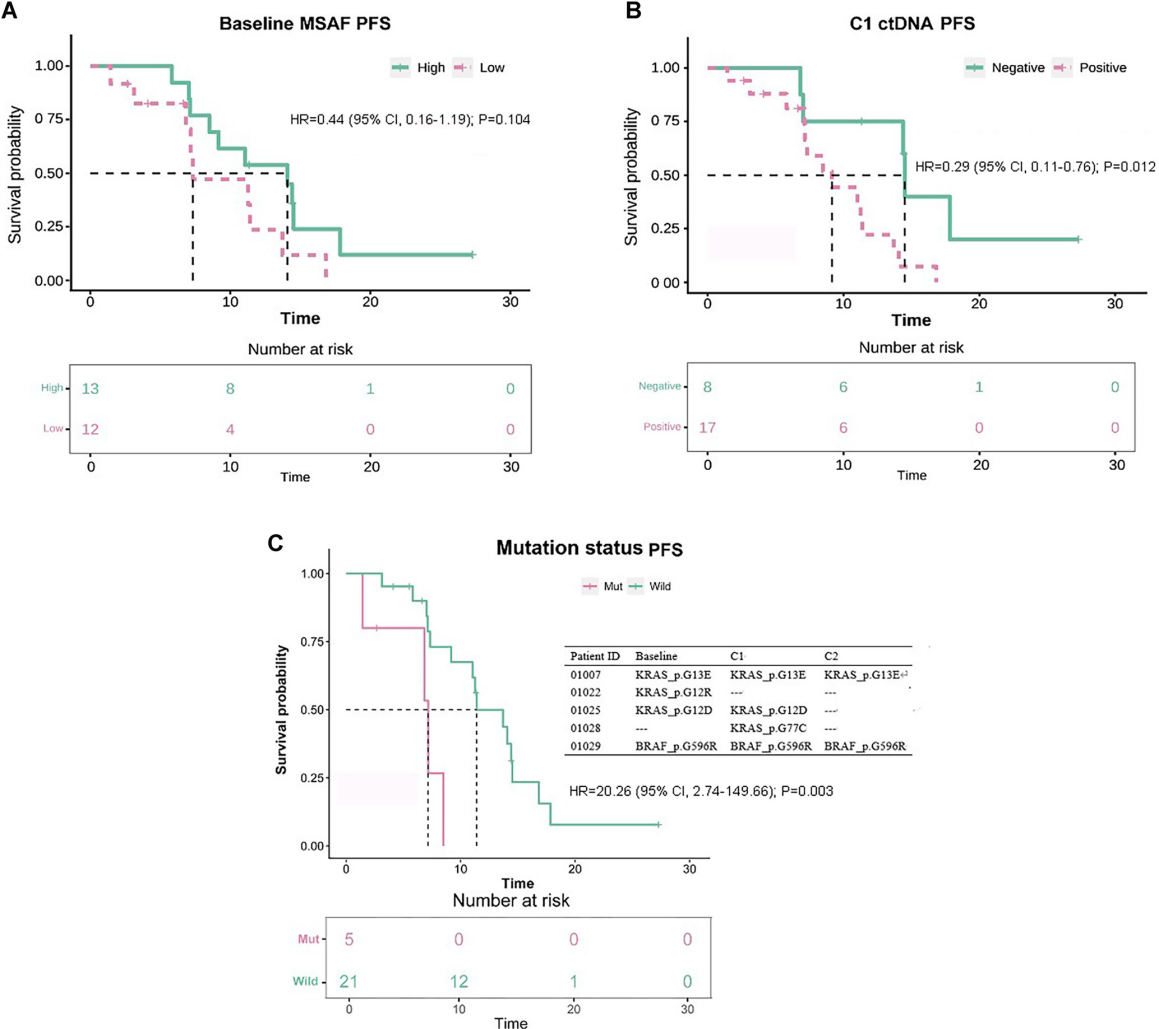
**Circulating tumor DNA metrics and progression-free survival.** Kaplan–Meier curves for PFS in patients receiving first-line anlotinib plus capecitabine and oxaliplatin, with numbers at risk shown below each plot. (A) PFS stratified by baseline ctDNA burden quantified as MSAF, dichotomized into high versus low using the cohort median cutoff (18.51%; *n* ═ 25). (B) PFS stratified by ctDNA status at C1 (timepoint near best radiologic response), defining ctDNA negativity as MSAF <0.001 (ctDNA-negative, *n* ═ 8; ctDNA-positive, *n* ═ 17; total *n* ═ 25). (C) PFS stratified by ctDNA-detected KRAS or BRAF mutation status (mutation detected vs wild-type; *n* ═ 26); the inset summarizes representative KRAS/BRAF variants detected across baseline, C1, and C2 timepoints in mutation-positive patients. HRs with 95% CIs are displayed on the plots; survival curves were compared using the log-rank test. Abbreviations: BRAF: B-Raf proto-oncogene, serine/threonine kinase; C1: Timepoint near best radiologic response; C2: Timepoint closest to radiologic progression or last follow-up; CI: Confidence interval; ctDNA: Circulating tumor DNA; HR: Hazard ratio; KRAS: Kirsten rat sarcoma viral oncogene homolog; MSAF: Maximum somatic allele frequency; PFS: Progression-free survival.

No significant difference in PFS was observed between patients with high vs low MSAF levels (14.06 [95% CI, 7.13–17.83] vs 7.33 [95% CI, 3.12–13.70] months, HR=0.44 [95% CI, 0.16–1.19]; *P ═* 0.104; Figure 5A), utilizing an MSAF cutoff value of 18.51%. Median PFS was significantly longer in ctDNA-negative patients compared to ctDNA-positive patients (14.52 [95% CI, 6.83–17.83] vs 9.17 [95% CI, 7.13–11.40] months; HR=0.29 [95% CI, 0.11–0.76]; *P ═* 0.012; Figure 5B) at the C1 timepoint. Furthermore, patients harboring mutations in *KRAS*/*BRAF* experienced shorter median PFS compared to wild-type patients (7.16 [95% CI, 1.45–8.50] vs 11.4 [95% CI, 7.33–14.52] months; HR=20.26 [95% CI, 2.74–149.66]; *P ═* 0.003; Figure 5C).

**Figure 6.**
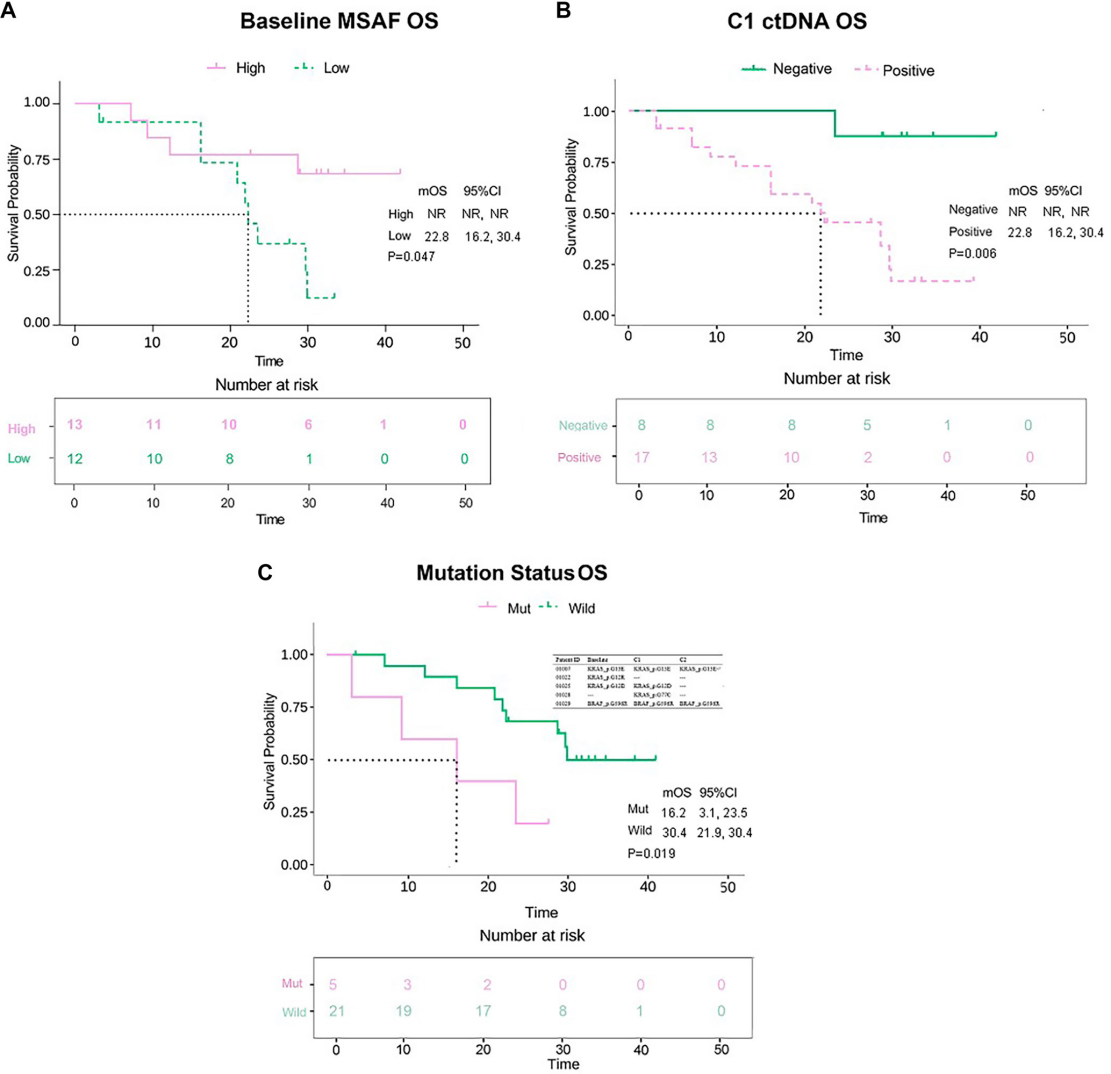
**Circulating tumor DNA metrics and overall survival.** Kaplan–Meier curves for OS in patients receiving first-line anlotinib plus capecitabine and oxaliplatin, with numbers at risk shown below each plot. (A) OS stratified by baseline ctDNA burden quantified as MSAF, dichotomized into high versus low using the cohort median cutoff (18.51%; *n* ═ 25). (B) OS stratified by ctDNA status at C1 (timepoint near best radiologic response), defining ctDNA negativity as MSAF <0.001 (ctDNA-negative, *n* ═ 8; ctDNA-positive, *n* ═ 17; total *n* ═ 25). (C) OS stratified by ctDNA-detected KRAS or BRAF mutation status (mutation detected vs wild-type; *n* ═ 26); the inset summarizes representative KRAS/BRAF variants detected across baseline, C1, and C2 timepoints in mutation-positive patients. HRs, mOS and 95% CIs are displayed on the plots; survival curves were compared using the log–rank test. NR indicates that the median OS was not reached at the time of analysis. Abbreviations: BRAF: B-Raf proto-oncogene, serine/threonine kinase; C1: Timepoint near best radiologic response; C2: Timepoint closest to radiologic progression or last follow-up; CI: Confidence interval; ctDNA: Circulating tumor DNA; HR: Hazard ratio; KRAS: Kirsten rat sarcoma viral oncogene homolog; mOS: Median overall survival; MSAF: Maximum somatic allele frequency; NR: Not reached; OS: Overall survival.

The impact of ctDNA on OS is illustrated in Figure 6. A significant difference in OS was observed between patients with high MSAF levels (median OS, NR) and those with low MSAF levels (median OS, 22.8 [95% CI, 16.2–30.4] months) at baseline (HR=0.32 [95% CI, 0.11–0.99]; *P ═* 0.047; Figure 6A). CtDNA-negative patients demonstrated significantly better OS than ctDNA-positive patients at the C1 timepoint (median OS, NR vs 22.8 [95% CI, 12.2–30.4] months; HR=0.21 [95% CI, 0.07–0.63]; *P ═* 0.006; Figure 6B). Additionally, a significant decrease in OS was noted for patients harboring mutations in *KRAS/BRAF* compared to those with wild-type (median OS, 16.2 [95% CI, 3.1–23.5] vs 30.4 [95% CI, 21.9–30.4] months; HR=8.42 [95% CI, 1.42–50.18]; *P ═* 0.019; Figure 6C). After multivariable Cox regression adjustment, ctDNA status at C1 was independently associated with survival (PFS, HR=0.10 [95% CI, 0.02–0.48], *P ═* 0.004; OS, HR=0.08 [95% CI, 0.01–0.71], *P ═* 0.023; Table S2).

## Discussion

This study explored the dynamic changes in ctDNA and their correlation with the treatment efficacy of first-line anlotinib plus CAPEOX in patients with mCRC. By employing mutation profiling of ctDNA, we have characterized the mutational landscape in response to combination therapy in mCRC. Furthermore, the on-treatment changes in ctDNA levels were associated with treatment response.

Consistent with previous studies, somatic mutations including *APC, KRAS, TP53, PIK3CA,* and *BRAF* were frequently identified in CRC [18, 20, 32]. Our findings align with these observations. Although all patients in our study were histologically or cytologically confirmed to be RAS/BRAF wild-type, we detected RAS/BRAF mutations in four individuals using ctDNA analysis. This discordance highlights the inherent heterogeneity between ctDNA-based detection and conventional diagnostic modalities [33, 34]. Studies examining BRAFV600E mutations in both tumor tissue and plasma have demonstrated that spatial heterogeneity may lead to the detection of BRAFV600E or other mutations (such as *KRAS* and *NRAS*) in plasma from patients who tested negative in tissue samples [35, 36]. These findings suggest that ctDNA analysis may capture the dynamic landscape of genetic alterations in cancer. Additionally, our analysis of baseline ctDNA samples revealed a significant association between ctDNA levels and metastasis site, particularly with liver metastasis, corroborating findings from other studies [37, 38]. Symonds EL *et al.* and Osumi H *et al.* demonstrated that patients with liver metastasis had higher ctDNA levels in plasma compared to those without [37, 38].

To further elucidate the clinical utility of ctDNA in mCRC progression, we assessed whether temporal changes in ctDNA levels during treatment correlate with radiologic progression. Data from other studies indicate that changes in ctDNA levels during CRC treatment correspond well with radiologic responses [19, 39]. Jia N *et al.* found that ctDNA levels could differentiate between patients with PD and non-PD responses in mCRC, showing that ctDNA levels decreased in the non-PD group and increased in the PD group over four treatment cycles [39]. Furthermore, Tie *et al.* demonstrated a positive correlation between changes in tumor size and ctDNA levels [19]. In line with these studies, our investigation found that MSAF levels at the C2 timepoint were elevated in both the non-PD and PD groups, with significantly higher levels in the PD group. Our findings also indicated that MSAF levels were notably higher in the PD group compared to the non-PD group at the C1 timepoint, suggesting a correlation between MSAF levels at C1 and tumor remission at C2. Collectively, our results provide preliminary evidence that ctDNA detected near the point of optimal remission may be associated with subsequent treatment outcomes; however, due to the limited sample size, these observations should be interpreted with caution and validated in future studies.

Another critical aspect of understanding the clinical value of cfDNA is its potential relationship with survival outcomes. Numerous studies support the clinical utility of ctDNA in impacting OS and PFS in mCRC [19, 20, 40–42]. Patients with RAS/KRAS mutations exhibit poorer survival rates compared to those without these mutations, as demonstrated in several clinical studies [43, 44]. While our findings align with this observation, the disparities in sample sizes between the RAS/KRAS mutation and wild-type groups necessitate cautious interpretation. Furthermore, our results indicate that ctDNA-positive patients experience significantly worse PFS and OS compared to ctDNA-negative patients, corroborating findings from other studies. McDuff et al. reported that postoperative ctDNA positivity was associated with significantly worse PFS in rectal cancer patients [45], while Lonardi et al. demonstrated that ctDNA-positive status at the time of PD or last follow-up correlated with a marked reduction in OS [42]. Additionally, the varying association patterns of baseline MSAF status with PFS and OS may be influenced by the timing of measurements, differences between PFS and OS endpoints, and the limited sample size. Baseline MSAF (high vs low) reflects intrinsic tumor burden and aggressiveness prior to treatment initiation and serves as a static indicator. Its impact on outcomes generally requires long-term follow-up, explaining the stronger association with OS than PFS. In contrast, ctDNA status reflects early treatment efficacy and correlates dynamically with short-term progression and OS. The limited sample size further complicates the detection of relationships between baseline MSAF and PFS. Given the exploratory nature of our study, larger studies are needed to validate the association of ctDNA at various time points with clinical outcomes.

Evidence from genomic studies and a small clinical study utilizing tissue biopsies suggests that patients with mutations in genes of the WNT pathway may exhibit primary resistance to oxaliplatin-based chemotherapy or targeted therapies [46, 47]. We identified activating mutations in the WNT pathway among patients with progressive disease, indicating the feasibility of identifying primary drug-resistant patients through ctDNA analysis. However, beyond the WNT pathway, the TP53 and Notch pathways are also associated with numerous genetic alterations, suggesting that establishing a definitive one-to-one relationship based on a single genetic alteration may be challenging. This aligns with findings from Shaib *et al.*, which indicate that multiple factors contribute to resistance in CRC, including ligand expression and activation of the phosphoinositide 3-kinase (PI3K) or insulin-like growth factor 1 (IGF-1) pathways [48]. A larger cohort study is warranted to further explore these potential mechanisms.

Several limitations of our study must be acknowledged. Firstly, the exploratory nature of the research, combined with a relatively small sample size and limited availability of plasma samples, may reduce the robustness and generalizability of our findings. Secondly, the inclusion of only two follow-up time points limits our ability to fully capture dynamic molecular changes during treatment. Thirdly, from an analytical perspective, KEGG pathway analyses were restricted to patients with PD, without assessing their relationship with clinical outcomes. Our dataset cannot clarify whether acquired mutations resulted from individual drugs or combination therapy. We only compared ctDNA features with baseline clinical factors; future studies could incorporate additional on-treatment circulating biomarkers to better evaluate their association with treatment response. Potential selection biases should also be considered. Excluding patients without C2 ctDNA data may introduce selection bias and affect the representativeness of our cohort. Additionally, the observed associations between C1 ctDNA status and survival outcomes should be interpreted with caution, as analyses may be influenced by immortal-time bias related to the duration required for patients to reach the C1 assessment. Lastly, from a statistical perspective, while continuous modeling of ctDNA variables is preferable in larger cohorts, dichotomization was utilized in this analysis due to sample size constraints. Although multiple longitudinal comparisons were corrected using the Bonferroni method for dynamic ctDNA changes, these results remain exploratory due to the small sample size. Furthermore, the small sample size limits the robustness of multivariable Cox regression analyses, necessitating cautious interpretation of the resulting hazard ratios. Validation in larger, prospectively designed cohorts with more frequent sampling is therefore warranted.

Given the preliminary and exploratory nature of our study, the relatively small sample size only permits associative conclusions between ctDNA features and clinical outcomes, and these findings should be regarded as exploratory rather than definitive. This limitation is partly due to methodological constraints related to biomarker testing. Previous studies have demonstrated that the lack of standardization in molecular testing—stemming from variations in gene panels, sequencing methods, and bioinformatics pipelines—leads to inconsistencies in biomarker detection and interpretation [49]. This challenge is evident in our study, where a mixture of fixed-panel and tumor-informed approaches likely introduced assay heterogeneity, potentially affecting analytical sensitivity. The mutational analysis of ctDNA via NGS must account for the possibility of false positives due to CH, as the presence of germline variants from normal cells poses limitations in data interpretation [50]. To mitigate this, matched WBC DNA was employed for both germline and clonal hematopoiesis of indeterminate potential (CHIP) filtering, and an in-house pipeline was utilized to eliminate likely CHIP-associated variants.

## Conclusion

The results of this post-hoc analysis suggest that dynamic changes in ctDNA may be associated with treatment response in patients with mCRC receiving anlotinib plus CAPEOX. These observations indicate that ctDNA may reflect tumor dynamics and evolving molecular changes during therapy, warranting further investigation.

## Supplemental data

Supplemental data are available at the following link: https://www.bjbms.org/ojs/index.php/bjbms/article/view/13678/4141.

## Data Availability

The datasets supporting the results of the present study can be obtained from the corresponding author upon reasonable request.
